# EU27 regional trade networks for medical products in fight against Covid-19 pandemic: Quantifying vulnerability and self sufficiency in critical inputs

**DOI:** 10.1371/journal.pone.0297748

**Published:** 2024-02-23

**Authors:** Sheri M. Markose, Semanur Soyyiğit

**Affiliations:** 1 Economic Department, University of Essex, Colchester, Essex, United Kingdom; 2 Department of Public Finance, Kirklareli University, Kirklareli, Türkiye; Villanova University, UNITED STATES

## Abstract

The Covid-19 pandemic spread fast due to a lack of vaccines and a severe shortage of medical products to treat and combat the disease. Many studies have focused on the characteristics of extant global supply chains and trade networks that are determined by globalization drives for production to low-cost countries and the technological complexity of products with many components distributed globally. This, along with the lockdown of many sectors and national policies that divert exports for domestic use, are reasons for lack of access, especially, in Western countries to these products. Governments adopted policies that aim to mitigate vulnerability to imports of critical medical products that include self-sufficiency measures such as increased domestic production, stockpiling and reduction of exports. However, there is as yet no quantitative way to assess if a country’s vulnerability to critical imports has been reduced by such drives for self-sufficiency, when other countries in the trade network follow similar policies. For this we develop a Google PageRank style centrality measure based on the Markose-Giansante eigen-pair method for a specially constructed global bilateral trade network to assess the vulnerability of net importers of critical medical products when they attempt to mitigate it by regional or domestic buffers. We use the network vulnerability centrality measure to quantify the regional self-sufficiency for EU27 countries over 2019–2021 for four Covid-19 critical medical products, viz. facemasks, personal protective equipment, ventilators and hand sanitizers. Despite, growth in value and share of intra-EU regional trade in most of these products by 2021, some countries did not achieve the reduction of vulnerability centrality.

## 1. Introduction

The Covid-19 pandemic, geo-political upheavals such as the Ukraine war and even policies to reduce greenhouse gas emissions have not just disrupted global supply chains but have called into question the viability of a policy of dependence on imports of critical products. A large number of studies, some of these will be reviewed, seek a framework to assess both future global trends in global supply chains and specific policy responses to enhance economic resilience [1–4] in the face of emergencies, which dictate the need to be self-sufficient in key areas such as medical supplies, food and energy. The vulnerability of countries and world markets for energy and foodgrains from recent Russian hostilities in Ukraine arise from their net export status in wheat and the Russian dominance in coal, oil and natural gas that account, respectively, for 18%, 11% and 20% of global exports [5].

Global Supply Chains (GSCs) and Global Value Chains (GVCs) are widely used concepts in the trade literature. GSCs refer to the cross-border organization of activities involved in the different stages of production of goods or services with their delivery by suppliers to consumers, respectively, in two different countries [6]. A global value chain is defined as the total of value-added activities by each country in the steps required to create a product or service from its conception, through design, sourcing raw materials and intermediate goods, production, marketing, distribution and support to final consumers [7]. Though the Covid-19 literature includes the tracking of GVCs, as in [2], since our data is based on import-export trade, it corresponds more with GSCs with the direct supply of a product or service from a supplier in one country to a customer in another.

While it is believed that a country’s participation in international trade and global supply chains is a significant determinant of economic development [8], there are now growing debates about their weaknesses of being organized globally and subject to national policy constraints [2,3,9–12]. Three global trends that predate the Covid-19 pandemic had started affecting GSCs and these trends are also bound to shape the future of GSCs [4]. The first of these trends is the tendency towards regionalization. Accordingly, growth of GSCs started slowing down following the Great Financial Crisis of 2007/8 and production became more regionalized. In contrast, globalization had accelerated with the acceptance of China to the World Trade Organization (WTO) in the early 2000s [13]. A decrease was observed in intra-regional trade in Europe and North America as inter-regional trade linkages with ‘Factory Asia’ became stronger [14]. The largest global companies started to outsource production and concentrate their foreign direct investment in China, specifically, and in Asia, generally. [15] explains this development to be a result of laissez-faire policies within the scope of Washington Consensus. The second trend, which is reversing offshoring of supply chains primarily to China, is the geopolitics behind the US-China trade war and their ‘high tech’ war [1,16] which, respectively purport, to rectify the disparities in the tariff structures between the two countries whilst they safeguard their technological superiority. Finally, the third trend that shapes GSCs is automation, digital transformation and artificial intelligence (AI), which have dematerialized production and retail, making “reshoring” as opposed to offshoring more feasible with micro-production and software services over the internet vitiating the advantages of low-cost labour supply in physical production [4].

Against this background, the Covid-19 pandemic has accelerated the discussion with the need to quantify the cost/benefits of GSCs and security issues in the supply of critical inputs such as medical equipment. These issues have galvanized countries to introduce more secure measures to increase resilience against global shocks. Breakdown of trust among the parties of the supply chain is one of the most important deficiencies of GSCs and it caused countries to question the reliability of GSCs. Covid-19 pandemic elicited interventionist policies by governments such as nationalisation of private hospitals in Spain, subsidy payments to companies to divert production for the national emergency, and even statements by France to nationalize large companies like car manufacturers suffering from Covid-19 lockdowns [17]. Apart from these country-specific implementations, there are also some debates related to regional policies. The EU implemented policies to increase the supply of medical products during the pandemic. These policies are [18]: (i) export bans, (ii) incentives to find new lines of production, (iii) some regulatory arrangements, (iv) diplomatic effort to prevent other countries’ export bans, (v) stockpiling. As similar policies also emanate from extra-EU countries, they have resulted in strategic onshoring drives in EU countries. One example is that of Italy, as the first European country to be hit by the virus, was rebuffed in their request to other European countries for help in supply of face masks [19].

The research question is on how to model and empirically assess the changing trade structure of the EU-27 region for pre-pandemic and pandemic periods in terms of its vulnerability and the efficacy of the adaptation measures undertaken to remedy the lack of self-reliance in critical medical products. We analyse this for four critical medical products that were found necessary [20,21] in the fight against the Covid-19 pandemic. These include facemasks, ventilators, personal protective equipment (PPE) and hand sanitizers. These four medical products have been identified to be those for which scarcity of supply followed from export restrictions by the largest number of countries [22]. In the face of the surge in demand [23], this led to the exacerbation of the medical emergency with intensive competition and bidding wars by countries that led to price spikes during the onset and peak of the pandemic.

We examine the intra-region trade network of the EU27 embedded in the global trade network for the four medical products in 2019, 2020 and 2021. These years cover, respectively, the pre-pandemic year, followed by the onset of Covid-19 pandemic in 2020 and the year 2021, which we call the ‘adaptation year’ while new cases are yet to peak. Different peak times are reported for different countries. COVID-19 cases peaked in January 2022 for the US, UK, Italy, Spain, France; February 2022 for the Netherlands; and March 2022 for Germany [24]. Hence, immediate, and time-lapsed responses of the countries to such an emergency will be analysed empirically and evaluated within the scope of regionalization and onshoring debates.

The lack of quantitative analyses, to evaluate the vulnerability of countries to import dependence in the critical medical products and how reshoring of their global supply chains and/or self-sufficiency policies sought from export restrictions to mitigate the medical emergency, is a glaring gap in much of the literature. We adopt cross border trade network analyses and centrality measures to determine the vulnerability of EU countries from their dependence on imports, especially from extra-EU countries. We use a Google PageRank style network centrality index developed in [25,26] in the context of vulnerability from financial contagion to quantify self-sufficiency or reduced vulnerability in critical medical products at the regional level of the EU27 countries. Network centrality measures are fixed point results which determine the relative position of a country in a manner that is consistent with other countries following similar strategies. For instance, a country by decreasing exports in critical products aims to increase its self-sufficiency, but in so doing can increase the vulnerability of other countries to which it has trade links. These indirect effects are captured recursively in a way that is consistent within the network as a whole. Thus, to quantitatively assess if overall countries have decreased their vulnerability requires a fixed-point algorithm provided by the Google PageRank style (see, [27,28]) principal eigenvector centrality associated with the maximum eigenvalue of a specially constructed matrix of net imports of countries adjusted for the regional self-sufficiency buffers. Finally, as part of the eigen-pair method of Markose-Giansante, the maximum eigenvalue gives information on the total systemic *ex ante* expected shortfall in net imports and the vulnerability centrality measure for each country apportions this to the individual country. Thus, the eigen-pair method, viz eigenvalue and eigenvector of the specially constructed net import matrix adjusted for buffers can ascertain the propensity and magnitude of shock cascades of the system vis-à-vis any of the participants of the network without having to conduct simulation exercises for the same.

Finally, in a general model where data on stockpiles/buffers of critical products is available, we draw attention to the comparable role that capital requirements play in financial contagion models to stabilize the system. Analogous to the epidemic R number for financial contagion [25], if the maximum eigenvalue of the specially constructed netted import matrix for the medical product adjusted by the value of the intra-regional trade is greater than 1, the ex-ante shortfall of net imports for that region will exceed its regional self-sufficiency measures by more than 100%. Our major contribution is to extend the global trade network models especially developed to examine the cascade effects of export restrictions by member countries for critical foodstuffs like wheat and other food grains (see, [29–31]). Note, such a global trade network framework for the Covid-19 critical medical products is almost entirely missing in discussions of the severe shortages faced by countries for these products.

Thus, we formulate a network centrality methodology that is general enough to track vulnerability of countries to global trade links while incorporating national thresholds for stockpiles and buffers of domestic production. However, due to the paucity of data on domestic production and stockpiles, and also policy variables regarding thresholds for the latter, we confine our analysis to the regional self-sufficiency centrality measures. For each of the medical products, we construct a bilateral net import matrix adjusted for the intraregional trade for the region that each country belongs to. This is to determine the extent to which regional self-sufficiency as proxied by the intra-regional trade is achieved. The latter indirectly reflects governmental policies, trade diversification and strategic substitution responses.

Based on this, the paper is structured as follows. **Section 2** gives a literature review. In **Section 3**, following a brief introduction to the information in the UN Comtrade database for the cross-border trade in the medical products for the pre-pandemic and pandemic years, we assess the adaptation year responses of 2021. **Section 4** introduces the relevant network methodology for network statistics for the intra-EU and global trade networks. This is followed by the Markose-Giansante eigen-pair method that is used to extend the Google PageRank style network centrality measures to quantify the vulnerability of EU27 countries from their import dependency on global trade networks for the four Covid-19 medical products. This enables us to see which of the EU27 countries succeeded with their regional self-sufficiency measures. The empirical results for this are given in **Sections 5** and **6**. Finally, we close with the concluding section.

## 2. Literature review

We primarily focus on studies relating to the role of GSCs for the critical medical supplies in exacerbating the Covid-19 emergency and also on those based on a quantitative network analyses of global trade structures. In regard to the latter, our paper contributes to the literature by showing how cascade models for food products are also applicable, with some extensions, to the case of Covid-19 medical products.

[32] speak of the ‘tragic failure’ of the lean management policies governing imports of critical products that led to inadequate national stockpiles when domestic demands soared at the height of the pandemic and exporting countries placed export restrictions. Regarding logistics within an economy, they propose a model that is more efficient in providing critical stockpiles and production by relying on new technologies such as advanced analytics and blockchain. The proposed model contains the following: A ‘National Supply Chain Command Centre’ which comprises three bases: (i) National Procurement Centre, (ii) National Supply Chain Quality Control Centre, (iii) National Distribution Centre. The second element is ‘Blockchain’ as a connector which assess the global healthcare supply chain through an ecosystem approach and provides an integrated view of the elements. ‘Predictive big data analytics in supply chain demand forecasting’ is the third element of the proposed model. [33] applied a stochastic optimization model to analyse the allocation of ventilators by Federal Emergency Management Agency (FEMA) to different US states. The results reveal that if less than 60% of ventilator inventories is available for non-Covid patients, then FEMA’s stockpile would be almost adequate to the needs. However, if more than 75% is available for non-Covid patients, then shortfall is expected in various degrees.

On the drawbacks of relying on global supply chains, the Asian Development Bank [34] underscores the risk of bottlenecks of the key materials, especially for PPE, in the fight against the virus, and emphasizes the importance of strengthening supply chains. By focusing on the interdependencies of trade of two essential categories of medical goods, [3] draws attention to administrative problems due to contentious interaction between the policymakers and leading manufacturers, and on how these problems increase the health risk for both medical personnel and patients. The author proposes some policy implications for the PPE case. [3] assesses how the international system has become more fragmented and regionally oriented in the aftermath of the Great Financial Crisis of 2008/09, with additional driving factors like the digital revolution, economic nationalism and the pandemic.

In their study, [35] emphasize how the pandemic has brought the vulnerability of the medical supply chain into focus and the systemic risks of reduced exports by major global producers to net importers of the medical products cannot be ignored anymore. [2] analysed the mutual constraints of both state policies and GVCs during the pandemic for PPE. The authors drew attention to two structural characteristics of GVC that countries interacted with: geographic scope and technological sophistication. The study pointed to these two as the key characteristics of GVCs in shaping the possibilities for state policy. These constraints were explicitly at work in the reversal of EU trade policies.

Reports from the OECD [36,37] emphasize the importance of trade in supplying the increasing demand for face masks, and the harm of export bans for the fight against the virus. However, [36] also emphasizes the deficiency of free trade and of export facilitation alone to solve the shortage of the key products during the fight against the pandemic. Accordingly, there should also be government planning and incentives for firms to adapt existing assembly lines to produce the needed medical product. [36] finds it costly for each country to build production facilities matching demand in the crisis period and proposes some upstream agreements with companies for rapid conversion of assembly lines during crises and some supportive international trade measures.

In addition to these qualitative studies and reports, the following empirical studies examine the global trade structure for medical goods by utilizing bilateral trade data between 35 reporting countries and 350 partner countries [38]. They use econometric analyses to examine the effect of political, economic, demographic and geographical ties on the bilateral trade of medical goods between countries. General results reveal that an increase in Covid-19 cases caused a decline in the export of the medical goods. However, this decline is lower when trade partners share some close political, economic and geographical ties. [39] examined the global trade network for facemasks and ventilators using the hub-authority centrality measure developed by [40] for the export matrix for over 200 countries. The top hub countries are net exporters with high centralities and top authority countries are net importers with high centralities. This approach is the closest to the one given in this paper and the authority countries are analogous to the principal eigenvector vulnerable net importer countries in our paper. [39] find that in the case of face masks, the top-ranking authority countries are almost exclusively developed countries, showing their vulnerability as dominant importers while no African country comes close. In the case of ventilators, the top-ranking hub centrality countries in 2019 was Singapore followed by Australia and China. In 2020, the surge in US production of ventilators, places the US as topmost hub central country followed by New Zealand and Germany. Interestingly, while this drive for self-sufficiency by the US, New Zealand and Germany made them the hub countries in 2020, the results in our paper show that US and Germany did not achieve the aimed for reduction in vulnerability.

Finally, as noted in the introduction, cascade effects in global trade network models have been used, for example, by [29–31], to quantitatively assess the vulnerability of countries to supply side shocks. These specifically include export restrictions in critical foodgrains like wheat. We will also follow [31,39] to use the methodology of [41] for the identification of the topological characteristics of a power law degree distribution in the global trade networks which signal hyper specialization of a few net exporters and to see if countries reduce their vulnerability as net importers by diversifying their import sources. The major contribution of our paper is to extend the analytical network results of these global trade models using insights from financial contagion models and their use of capital buffers to mitigate cascade failure. We use the framework from [25,42] to overcome the drawback of the network-based cascade models in economics in that they do not fully develop the equivalent of the R-number, well known from epidemic models to identify the stability of the networked system.

## 3. Overview of global trade data for 4 medical products relating to EU27 countries

### 3.1 2019 Pre-pandemic and 2020 pandemic years

We start with **Table 1**, which gives the description of the medical products as given by the World Customs Organization and the UN Comtrade data on global imports and that of EU27 countries for the 4 medical products, facemask, ventilators, PPE and sanitizer. The data was collected from the United Nations Comtrade database following the Harmonized System (HS) classification for Covid-19 medical supplies which was updated by [43] jointly with the World Health Organization.

**Table 1:** Description of the data for key medical supplies: World and EU27 import values ($ Billions) Pre-pandemic (2019) and pandemic (2020) years.

HS 2017 Classification	Name of the Medical Product	Description of the Medical Product	World import in current US Dollar (Billions)		EU27 import in current US Dollar (Billions)	
			2019	2020	2019	2020
6307.9	Face mask	Textile face masks, without a replaceable filter or mechanical parts, including surgical masks and disposable face-masks made of non-woven textiles. This includes the masks known as N95 Particulate Respirators	12.898	76.194	3.809	24.618
9019.2	Ventilator	Medical ventilators (artificial respiration apparatus)	8.054	15.055	2.426	2.633
4818.5	Personal Protective Equipment (PPE)	Paper or cellulose garments and clothing accessories such as disposable paper hospital gowns, paper shoe covers etc.	0.166	0.637	0.065	0.088
3808.94	Sanitizer	A liquid or gel generally used to decrease infectious agents on the hands, alcohol-based type	2.386	6.392	0.862	1.864
Total world import values			23.481	98.031	7.161	29.203

Source: We follow classification announced by [43] for the description of the medical products. The values in the Table 1 are calculated by the authors utilising the UN Comtrade database. HS: Harmonized System.

**Table 2** then proceeds to give the breakdown of shares of EU27 country imports from extra-EU and intra-EU sources of these medical products. **Table 3** identifies the top 5 extra-EU and intra-EU exporting countries to the EU27 countries. The data shows that increased demands for these medical products globally with the onset of the Covid-19 pandemic elicited different cross country trade outcomes depending on the nature of the global supply chains in situ that reflects the way in which production is distributed globally, determined in part by the technological complexity of the product [2,44]. In this section data has been analysed for the pre-pandemic year 2019 and the year 2020 when Covid-19 became a pandemic affecting multiple countries.

**Table 2:** Medical goods imported by EU27 $ (billions) broken down by extra -EU and Intra-UK (% of Total EU Imports), 2019–2020.

Medical Product	Extra-EU Import Values		Intra-EU Import Values		Total EU Import Values	
	2019	2020	2019	2020	2019	2020
Facemask	1.913	19.672	1.895	4.946	3.809	24.618
	(50.2%)	(79.9%)	(49.8%)	(20.1%)		
Ventilator	1.305	1.437	1.120	1.196	2.426	2.633
	(53.8%)	(54.6%)	(46.2%)	(45.4%)		
PPE	0.029	0.037	0.035	0.050	0.065	0.088
	(45.4%)	(42.5%)	(54.6%)	(57.5%)		
Sanitizer	0.136	0.407	0.726	1.457	0.862	1.864
	(15.7%)	(21.8%)	(84.3%)	(78.2%)		

Source: Calculated by the authors using the UN Comtrade Database. Note that the percentage values in the parenthesis is in respect to the total value of the EU imports.

**Table 3:** Import values for EU27 of medical products from Top 5 extra-EU and intra-EU Exports (current US dollar billions; *% of total EU27 Imports for each product), 2019–2020.

Extra-EU Top 5 exporter countries in EU27’s total imports						
Medical Products	2019			2020		
	Country	Billion US $	Market share* (%)	Country	Billion US $	Market share* (%)
Face mask	China	0.9597	25.2	China	17.314	70.3
	Morocco	0.1956	5.1	Vietnam	0.5053	2.1
	Vietnam	0.1444	3.8	United Kingdom	0.3531	1.4
	United Kingdom	0.1389	3.6	Turkey	0.3144	1.3
	Tunisia	0.0998	2.6	Hong Kong	0.277	1.1
	Top 5 Total	1.5384	40.4	Top 5 Total	18.7638	76.2
Ventilator	Singapore	0.2809	11.6	USA	0.3888	14.8
	USA	0.2731	11.3	Singapore	0.3031	11.5
	United Kingdom	0.2218	9.1	New Zealand	0.221	8.4
	Australia	0.2093	8.6	United Kingdom	0.2056	7.8
	China	0.108	4.5	Switzerland	0.1356	5.2
	TOTAL	1.0931	45.1	TOTAL	1.2541	47.6
PPE	Thailand	0.0239	36.9	Thailand	0.0306	35
	USA	0.0021	3.3	United Kingdom	0.0031	3.5
	China	0.0018	2.8	China	0.0018	2.1
	United Kingdom	0.0013	2.1	USA	0.0008	1
	Norway	0	0	Singapore	0.0005	0.5
	Top 5 Total	0.0292	45.2	Top 5 Total	0.0367	42
Sanitizer	United Kingdom	0.0953	11.1	United Kingdom	0.1683	9
	China	0.0175	2	China	0.0966	5.2
	USA	0.0171	2	Turkey	0.0933	5
	Norway	0.0021	0.2	USA	0.027	1.4
	Turkey	0.0012	0.1	Norway	0.0054	0.3
	Top 5 Total	0.1333	15.5	Top 5 Total	0.3906	21
Intra-EU Top 5 exporter countries in EU27’s total import						
Medical products	2019			2020		
	Country	Billion US $	Market share* (%)	Country	Billion US $	Market share* (%)
Face mask	Germany	0.5864	15.4	Germany	1.4741	6
	Netherlands	0.2529	6.6	Netherlands	0.5824	2.4
	Poland	0.1847	4.8	Poland	0.3423	1.4
	France	0.1592	4.2	Austria	0.3084	1.3
	Romania	0.1484	3.9	France	0.3025	1.2
	Top 5 Total	1.3317	34.96	Top 5 Total	3.0097	12.2
Ventilator	Netherlands	0.4155	17.1	Netherlands	0.5106	19.4
	Germany	0.2528	10.4	Germany	0.4679	17.8
	France	0.0962	4	Ireland	0.1465	5.6
	Czechia	0.0946	3.9	Denmark	0.0196	0.7
	Sweden	0.066	2.7	Spain	0.0182	0.7
	TOTAL	0.9251	38.1	TOTAL	1.1627	44.2
PPE	Netherlands	0.0089	13.8	Netherlands	0.0105	12
	Italy	0.0055	8.5	France	0.0072	8.2
	Germany	0.0047	7.2	Germany	0.007	8
	France	0.0038	5.8	Italy	0.0056	6.3
	Portugal	0.0035	5.3	Sweden	0.0039	4.5
	Top 5 Total	0.0264	40.8	Top 5 Total	0.0341	39
Sanitizer	Germany	0.2585	30	Germany	0.4453	23.9
	Belgium	0.0904	10.5	Spain	0.192	10.3
	Spain	0.0851	9.9	France	0.138	7.4
	France	0.0724	8.4	Belgium	0.1286	6.9
	Netherlands	0.0668	7.8	Netherlands	0.1172	6.3
	Top 5 Total	0.5732	66.5	Top 5 Total	1.0211	54.8

Source: Calculated by the authors using the UN Comtrade Database.

Before getting into the details, we will summarize the different global trade responses for the 4 medical products at the onset of Covid-19. For face masks, global and EU imports remained plugged into the extra-EU global supply chain to meet increased demands triggered by Covid-19 in 2020 and the dominance of China as global exporter of face masks increased. In value terms, face masks saw the largest increase of imports both globally and intra-EU, with intra-EU share of total EU imports suffering a fall from 49.8% to 20.1% as shown in **Table 2**. In the case of ventilators, while global imports grew by 87%, EU27 imports of ventilators both from extra-EU and intra-EU remain almost static in 2020 at about the 45% mark. Interestingly, **Table 2** shows that in the case of PPE and hand sanitizer, the EU27 was even in the pre-pandemic year of 2019 the major supplier to this regional market, respectively, accounting for, respectively, 54.6% and 84.3% in 2019. In 2020, in the case of PPE, the intra-EU share increased to 57.5% in 2020, but that of hand sanitizer fell to 78.2%.

### Face masks

Despite the export restrictions and global supply chain disruptions from Covid-19 lockdowns, we observe in **Table 1** that globally face mask imports increase almost 6 times from 2019 to 2020. In contrast, this reliance on global supply chains increases almost 8 times for the EU27 countries. This is underscored by **Table 2** which shows that in the case of the increased demand for face masks by EU27 countries, this was met by imports from extra-EU countries. A 10-fold increase is observed for face mask imports from extra-EU countries while this increase was limited to about 2.5 times in intra-EU trade. In fact, the immediate impact was that intra-EU share of imports by EU27 for facemasks fell from 49.8% in 2019 to 20.1% in 2020 of the total EU imports. This is despite intra-EU trade in face masks in 2020 increased to $4.9 bn from $1.8 bn in 2019. From **Table 3**, it is observed that Germany is the main provider of face masks to the region before the pandemic. However, there is a severe decline in the German export share of face masks to EU-27 in 2020 indicated diversion for domestic use. The other important providers in 2019 namely the Netherlands, Poland and France also have decreasing share in intra-EU import of face masks by 2020. As noted by [45] **Table 3** shows the dominance of China as the most important provider of face masks in the pandemic year. China’s share in extra-EU import increased from 25.2% in 2019 to 70.3% in 2020. In 2019 Morocco had a 5% share in exports to EU27 with Vietnam at third place with 3.8% share. In 2020, the massive expansion by China leaves other extra-EU exporters for face masks being squeezed out with UK taking 3^rd^ place with a small share of 1.4%.

### Medical ventilators

The global increase from 2019 to 2020 in imports of ventilators is 87% and for EU27 is only 9%. **Table 2** shows that imports by EU27 from extra-EU countries of ventilators in 2019 is 53.8% and in 2020 this grows very little to 54.6%. Intra-EU share of exports to EU27 is 46.2% in 2019 and this falls to 45.4% in 2020. **Table 3** shows Netherlands is the dominant intra-EU exporter in both years and also increasing its market share from 17.1% to 19.4%. Germany is at 2^nd^ place in intra-EU exports and it increases market share from 10.4% in 2019 to 17.8% in 2020 at the expense of France. Note the dominant extra-EU exporter to EU27 countries is Singapore with market share of 11.6% in 2019, followed by the US and UK with respective market shares of 11.3% and 9.1%. In 2020, the US overtakes Singapore with market share of 14.8% and New Zealand takes 3^rd^ place from UK.

Among the medical products, bandages, PPEs, face masks, safety glasses and medical gloves have limited complex structure when compared to the more technically complex components needed for ventilators [45]. Ventilators consist of many components including electronic chips. Thus, increased domestic production of ventilators, whether for the domestic market or for exports, requires the procurement of a number of complex components all at once [44]. Automobile manufacturers in the US such as General Motors and Ford shut down their production lines due to the pandemic in the beginning of March in 2020. Then, they adapted their production lines to produce medical ventilators [44]. When it comes to the EU, major European firms contributed to 60% of global ventilator market, implying that know-how and production capability is available in the EU. However, input suppliers of these firms are scattered all over the world. Besides, low automation and difficulties in hiring labour with proper capabilities were short-run obstacles in the increased production [45]. Last but not least, closure of chip factories in Asia caused delays accessing this vital component in the production process. Hence, single sourcing was another difficulty [18]. The effect of these difficulties is observed in **Table 2** in the short term, which seems to have been overcome by 2021 as will be seen in **Table 5** where the intra-EU share of total EU27 imports for ventilators has grown to 50.9% from the 45% mark in 2019 and 2020.

#### PPE

**Table 1** shows an almost 4-fold increase in global imports for PPE in 2020. The EU27 imports in PPE grew by 35%. **Table 2** shows that even in the pre-pandemic year of 2019, by far, intra-EU imports of PPE account for the dominant market share for EU27 total imports at 54.6%. Indeed, extra-EU imports of PPE by EU27 fell from 45.4% in 2019 to 42.5% in 2020 and in 2021 it fell further to 32.3% of total EU27 imports. In this period over 2019 to 2020, **Table 2** indicates that the intra-EU share of EU27 imports for PPE grows from 54.6% to 57.5%, with **Table 3** showing that Netherlands had dominant share of 13.8% in 2019 and France and Germany increase their market shares in 2020. **Table 3** shows that Thailand is the dominant exporter of PPE in both 2019 (36.9% share) and 2020 (35%). US (3.3%), China (2.9%) and UK (2.1%) made small export contributions in 2019 to EU27 imports of PPE. In 2020, none of these countries make any headway as exporters of PPE. [2] discusses the negative impact beggar thy neighbour trade restrictions imposed by governments in the export of nationally required protective and disposable clothing which led to widespread domestic shortages in Europe, US and UK. Further, global supply chain shortages in the raw materials such as polypropylene which is melted to produce the nonwoven melt-blown fabric for PPE has been cited by [44] as bottlenecks in expanding production of PPE to the extent needed to meet the Covid-19 demands. This is despite intra-EU share of imports rising to 57.5% in 2020 and to 67.7% in 2021. Globally, Thailand is the top provider of PPE in both years, however its share is decreasing from about 37% in 2019 to 35% in 2020. The US and China also have decreasing shares between these two years.

#### Hand sanitizers

When it comes to sanitizers, the EU27 are not majorly dependent on extra-EU countries with the share of the latter being around only 15.7% in 2019. This grows to 21.8% in 2020 and UK is the dominant extra-EU exporter to the EU27. Spain also contributes a 10% share to intra-EU imports of sanitizer and all EU countries register a small increase in share of intra-EU imports in 2020. The explanation for a relatively low extra-EU dependence for sanitizers lies in the fact that leading market players in this product are European companies such as Reckitt Benckiser, Procter & Gamble, GOJO Industries, Cleenol, EO products etc. in a region of the world with very high hygiene standards [46]. Further, expansion of production is easily accomplished with distilling and bottling units having the infrastructure to switch production to sanitizers in dispensers. **Table 5** shows that for sanitizer, the top two exporters within the region namely Germany and Belgium have a decreasing share in intra-EU import in the pandemic year. Spain has an increasing share in intra-EU import from 2019 to 2020. Nevertheless, **Table 2** shows that there was a small fall in the intra-EU share of imports of hand sanitizers by EU27 from 84.3% to 78.2%, which remains almost static at 77.5% in 2021. For sanitizer, the top two exporters within the region, namely, Germany and Belgium have a decreasing share in intra-EU import in the pandemic year. Spain has an increasing share in intra-EU import from 2019 to 2020.

In conclusion, we find that in the case of face masks, EU-27 dependence in 2020 on extra-EU imports primarily from China increases to 70.3% of total EU imports. While EU-27 had a high degree of self-sufficiency for hand sanitizers throughout the period, increasing EU-27 this for PPE and medical ventilators was hampered by the relative complexity of their production process. In PPE some headway was made with intra-EU imports rising to 67.7% in 2021. In medical ventilators, there was only a 5% increase.

### 3.2 Adaptation year 2021 to Covid-19 pandemic: Was there regional self-sufficiency?

Thus far, we observed that immediate increase of demand in medical goods that came about with the spread of Covid-19 in 2020 created a somewhat chaotic and varied response in the different countries given the geographically uneven distribution of production of the medical goods and the national policies being pursued. **Table 4** shows how the world import values of 2021 compare with 2020 from **Table 1**. It is seen that there is a severe decline of the import of these medical goods by the EU27. Reports suggest that the peak of Covid-19 in terms of new cases was reached in January 2022 or later for most of the Western countries in this study. Thus, the fall in world imports of medical products in 2021 can be taken to be a sign of domestic self-sufficiency strategies including the restriction of exports rather than the case that the demand for these products has fallen.

**Table 4:** World and EU27 import values ($ Billions) for key medical supplies, 2021.

Medical Product	World import in US Dollar (Billions)	Change from 2020 to 2021 (%)	Total EU27 import in current US Dollar (Billions)	Change from 2020 to 2021 Total EU27 imports (%)
Face mask	24.400	-67.98	7.744	-68.54
Ventilator	12.273	-18.48	3.047	15.74
PPE	0.209	-67.14	0.065	-26.15
Sanitizer	4.216	-34.05	1.370	-26.50
TOTAL	41.098		12.226	

Source: Author’s own calculation by utilising UN Comtrade Database. 2020 values are given in **Table 1**.

Thus, in terms of global imports, **Table 4** shows that in 2021 there is a 67% decrease for face mask and PPE, 34% decrease for sanitizer and 18% decrease for ventilator. The case is a bit different for the EU27 imports. There is a 68% decrease in total imports for EU27 for face masks from 2020 to 2021 mirroring the global trade, while there is much smaller decrease at the EU level at about 26% for PPE and sanitizer. However, EU ventilator imports exhibit 15% increase from 2020 to 2021. Note, in the absence of country level domestic production data for the medical products, in the case of EU27, we will rely on the growth of the share of intra-regional imports to total EU imports to indicate regional self-sufficiency.

Regarding **Table 5**, the last column shows how in 2021, with the exception of hand sanitizer, intra-EU share of EU27 total imports grew substantially by 128.86% for face mask, 12.11% for ventilator and 17.74% for PPE. This gives some evidence for regional self-sufficiency in the EU27 countries for these medical products in the adaptation year.

**Table 5:** Medical goods imported by EU27 $ (billions) (% of Total EU Imports of the product), 2021.

Medical Product	Extra-EU Import Values $ bns (%)	Intra-EU Import Values $ bns (%)	Total EU Import Values $ bns	% Change in Intra-EU share of Total EU27 Imports
	2021	2021	2021	2020–2021
Face mask	4.183	3.561	7.744	128.86
	(54%)	(46%)		
Ventilator	1.495	1.553	3.047	12.11
	(49.1%)	(50.9%)		
PPE	0.021	0.044	0.065	17.74
	(32.3%)	(67.7%)		
Sanitizer	0.308	1.062	1.370	-0.90
	(22.5%)	(77.5%)		

Source: Author’s own calculation by utilising UN Comtrade Database.

When we compare **Table 5** with **Table 2**, we observe that the increased extra-EU share in total EU27 imports with the pandemic in 2020 shows a reversal in 2021 for face masks and ventilators, meaning that the region became more self-sufficient in the adaptation year. However, the region still gets more than half of its face mask imports and almost half of its ventilator imports from extra-EU countries. In terms of PPE, there is a small fall in share of the extra-EU imports and high percentage of total import of the region at 67.7% is provided from intra-EU. Although the share of extra-EU in total sanitizer imports increases by about 1%, the percentage share of intra-EU import to total EU27 imports at 77.5% is very high for this product. By looking at these percentage values, we can deduce that sanitizer and PPE are medical products for which the region provides the highest self-sufficiency.

Evaluating **Table 6** together with the **Table 3**, it can be said that Asian countries like China and Thailand, respectively, are still major extra-EU trade partners for face mask and PPE despite decreasing percentages in the adaptation year 2021. Another important result from this evaluation is that Germany and the Netherlands are two major supplier countries of face mask, PPE and sanitizer with increasing percentage shares in intra-EU trade.

**Table 6:** 2021 adaptation year import values for EU27 of medical products from Top 5 extra-EU Countries (current US dollar billions; *% of total EU27 Imports for each product).

Medical products	Extra-EU Top 5 exporters in EU27’s import			Intra-EU Top 5 exporters in EU27’s import		
	Country	Billion US $	%*	Country	Billion US $	%*
Face mask	CHN	3.0486	39.4	DEU	1.0382	13.4
	TUR	0.2972	3.8	NLD	0.5107	6.6
	GBR	0.1789	2.3	POL	0.3521	4.5
	TUN	0.1468	1.9	FRA	0.3152	4.1
	MAR	0.1210	1.6	ROU	0.1941	2.5
	TOTAL	3.7925	49.0	TOTAL	2.4104	31.13
Ventilator	USA	0.3719	12.2	NLD	0.5470	18.0
	NZL	0.3314	10.9	DEU	0.3783	12.4
	SGP	0.2242	7.4	FRA	0.1433	4.7
	GBR	0.1747	5.7	CZE	0.1223	4.0
	AUS	0.1621	5.3	ITA	0.0923	3.0
	TOTAL	1.2642	41.49	TOTAL	1.2832	42.11
PPE	THA	0.0171	26.5	NLD	0.0153	23.7
	CHN	0.0015	2.4	DEU	0.0062	9.6
	GBR	0.0010	1.6	FRA	0.0051	7.9
	USA	0.0010	1.5	ITA	0.0035	5.4
	MYS	0.0001	0.1	PRT	0.0031	4.8
	TOTAL	0.0207	32.10	TOTAL	0.0332	51.43
Sanitizer	GBR	0.1497	10.9	DEU	0.3196	23.3
	CHE	0.0792	5.8	NLD	0.1432	10.5
	CHN	0.0379	2.8	ESP	0.1418	10.4
	USA	0.0223	1.6	FRA	0.0988	7.2
	TUR	0.0104	0.8	BEL	0.0828	6.0
	TOTAL	0.2995	21.8571	TOTAL	0.7862	57.3863

Source: Author’s own calculation by utilising UN Comtrade Database.

## 4. Global trade network methodology for topology and stability

### 4.1 Network statistics

In this section we will discuss network statistics for the global trade networks involving EU-27 countries and for the intra-EU trade networks for the 4 medical products. The aim is to see how the topology of these networks changed from the 2019 pre-pandemic year and in the years 2020 and 2021.

A network is defined as *G = (V*,*E*,*f)*, where *V* is a finite set of nodes, *E* is the set of connections between these nodes, and *f* is a mapping that connects the elements of *E* with the elements of *V* [47]. The major mathematical tool for the quantitative analyses of networks is a matrix with dynamics of such systems captured by the power iteration of matrices. The matrix, called the adjacency matrix, encapsulates the binary relationship of any two nodes of a network as follows [47]:

$$A_{ij}=\{\begin{array}{cc} 1 & \mathrm{if}\;i,j\in E\\ 0 & \mathrm{otherwise}. \end{array}$$ (1)

In weighted networks, the weights of the links between node pairs constitute the elements of the matrix.

Thus, the global trade networks are weighted directed graphs and will be based on bilateral import matrices using UN Comtrade data described in **Table 1.** Here, the gross import matrix for each product, ***X***, has elements, *X*_*ij*_, which denotes what country *i* imports from country *j*. N is the total number of countries in a given trade network.


$$X=[\begin{array}{cccccc} 0 & X_{12} & \cdots & X_{1j} & \cdots & X_{1N}\\ X_{21} & 0 & \cdots & X_{2j} & \cdots & X_{2N}\\ \vdots & \vdots & 0 & \cdots & \cdots & \cdots \\ X_{i1} & \vdots & \cdots & 0 & \cdots & X_{iN}\\ \vdots & \vdots & \cdots & \cdots & 0 & \vdots \\ X_{N1} & X_{N2} & \cdots & X_{Nj} & \cdots & 0 \end{array}]$$ (2)

The sum of i-th row, $\sum_{j=1}^{N}X_{ij}$, corresponds to total imports of country i, while the sum of j-th column, $\sum_{i=1}^{N}X_{ij}$ corresponds to total exports of country j. Note, the transpose of the matrix in (2) gives the global export trade matrix.

The first step in complex network analysis involves the topological features using a set of informative measurements [48], typically relating to connectivity, clustering, reciprocity, degree distribution, assortativity/disassortativity and centrality.

Connectivity is measured locally by the quantity which is called ‘strength’ in weighted networks. Strength reveals the weights of the out degrees from country *i* to all the *j* countries from which country *i* imports. Besides this local measure of connectivity, there is also a non-local measure that represents connectivity for overall network [49]. Density measure is a global measure that represents the connectivity for overall network. The formula of density for a directed graph *G* is:

$$\delta (G)=\frac{m}{N(N-1)}=\frac{\bar{k}}{N-1}$$ (3)

where *m*, the total number of links, is divided by the maximum possible number of links in the network given by the product of total number of nodes *N* and their connection to other nodes, *N-1* [50]. Here, $\bar{k}=\frac{m}{N}$ is the average number of number of links (out degrees) per node. Graph *G* is called sparse when $\bar{k}\ll N-1$, while it is called dense when $\bar{k}\approx N-1$ [51].

Clustering coefficient, which is a local measure defined for each node *i*, is interpreted as the probability of two nearest neighbours of node *i*, which are also connected to each other. $C_{i}=\frac{2\Delta_{i}}{k_{i}(k_{i}-1)}$ is the formula of clustering coefficient for node *i* where *k*_*i*_ is the number of *i’s* neighbours [52]. Clustering coefficient of the overall network is calculated by averaging of all *C*_*i*_ values. Hence, the clustering coefficient for overall network lies between 0 and 1: 0≤*C*≤1. *C* = 0 if all neighbors are unconnected for all nodes and *C* = 1 if all nodes are connected to one another [53]. Reciprocity in a directed network measures the probability of having edges in both directions between any two vertices. In economic terms, it indicates how much two economies are interconnected or how much one economy depends on the other to fulfil its needs. Reciprocity ratio is formulised as $r=\frac{L^{↔}}{L}$, where *L*^↔^ refers to the number of reciprocal links and *L* refers to the total number of links. Reciprocity ratio lies between 0 and 1. If *r* = 0, then there is no reciprocity, and if *r* = 1, then all links are reciprocated in the network [49].

Degree distribution of a network can characterize ‘complexity’ in accordance with the presence of scale-free distribution (or fat tails) [49]. In degree distribution of a network, scale-free structure implies a power-law distribution, [41] which is defined as *P*(*k*)∝*k*^−*α*^, with *α* as the power law exponent. If *k* is the number of in-degrees of a node (signalling its export status in an import trade matrix), the smaller the α, higher the probability, *P(k)*, of observing nodes with very large *k*. In other words, a network with a lower value of *α* has a higher probability of hyperconnected nodes in comparison with a network with a higher value of α [54]. Thus, power law distribution indicates the existence of superhubs, which in the case of trade networks implies overspecialization of a few countries as net exporters with many countries importing from a few. Trade diversification by net importers is a means by which countries can reduce their vulnerability. That is why, it is important in a topological analysis of a global trade network to examine if degree distribution follows a power-law or not. While skewness and kurtosis values useful to describe the degree distribution, it is now customary to use the method proposed in [41] which combines maximum-likelihood fitting methods with Kolmogorov-Smirnov (KS) goodness-of-fit tests for detecting empirical power law distribution. Finally, a power-law distribution shows that network system is directed by a few core nodes with high degree/strength. These core nodes determine the dynamic stability of the whole network even if they are quite few [55].

Degree-degree correlation indicates the way in which nodes are connected in a network. Specifically, a network in which nodes with high-degree tend to connect to one another has positive degree-degree correlation and this kind of networks are called ‘assortative networks’. On the other hand, a network in which nodes with high-degree tend to be connected to low-degree nodes have negative degree-degree correlations and this kind of networks are called ‘disassortative networks’. A way of determining this structure is by measuring correlation coefficient of this degree-degree correlation [50]. If this correlation coefficient is higher than 0, then the network is assortative, and if the coefficient is lower than 0, then the network is disassortative. An assortative structure reveals that hubs are mostly connected to hubs, while a disassortative structure reveals that hubs are mostly connected to small-degree nodes in the network [53]. Therefore, disassortative structure confirms the presence of a core-periphery structure in a network [56,57]. Such structures are well known to characterize global trade and financial networks.

### 4.2 Network centrality and regional self-sufficiency index: Eigen-pair method

Here we follow [25] eigen-pair network centrality and stability model, which was first developed in [42] for financial contagion analysis and to identify systemically important countries or firms and those that are vulnerable from financial exposure to the former. Likewise, in our analysis of trade networks for the Covid-19 pandemic we need to quantify the degree of vulnerability of a country due to its dependence on imports for key medical products. The notion of self-sufficiency will be considered to be inversely related to the vulnerability centrality index, viz. higher this index, the lower the self-sufficiency. The model has scope to incorporate domestic stockpiles as buffers and thresholds can be defined for these buffers that can be used to offset shortfalls in imports which will adversely affect the size of stockpiles. Denoting initial stockpiles for the medical product in country *i* as *S*_*i0*,_ at each time step, it is envisaged that rates of vulnerability denoted as, *μ*_*it*_, is given by:

$$\mu_{it}=\frac{S_{i0}-S_{it}}{S_{i0}}.$$ (4)

At any *t*, *S*_*it*_ can be zero when all of the stockpile has been depleted and the *μ*_*it*_ index can shoot up to 1, indicating a 100% loss. This drives inventory control policies such that *S*_*it*_>*ρ*_*i*_ *S*_*i*0_ and *ρ*_*i*_ *S*_*i*0_ is the lower bound to which the stockpile can go to. Hence, it can be assumed that at each time step the cumulative loss is given by (*S*_*i*0_−*S*_*it*_) and net loss after mitigation is given by (*S*_*i*0_−*S*_*it*_) (1−*ρ*_*i*_). Borrowing from the epidemic literature *ρ*_*i*_, 0 ≤*ρ*_*i*_<1, is taken to be ‘cure rate’, and so (1−*ρ*_*i*_) is the rate of not recovering. The size of a country’s stockpile *S*_*it*_ is assumed to be adversely affected by the expected shortfall in net imports for a country *i*, where the shortfall arises from a similar vulnerability rate for the net exporter countries *j*. Thus,

$$S_{it+1}=S_{i0}-\sum_{j}(X_{ij}-X_{ji}{)}^{+}\mu_{jt}-(1-\rho_{i})(S_{i0}-S_{it}).$$ (5)

In (5), the vulnerability, *μ*_*jt*_, of country *i’s* net exporter trading partners *j*, where those with *μ*_*jt*_>0 and closer to 1 imply drastic shortfalls in net imports made by country *i* from country *j*. This increases the vulnerability of country *i* as stockpile at *t+1*, *S*_*it*+1_, goes to zero faster depending on the size of *i’s* exposure to counterparty *j* and on how vulnerable *j* is. In (5), the superscript + in the term (*X*_*ij*_−*X*_*ji*_)^+^ denotes that country *i* is a net importer and only (*i*,*j*) pairs are included for which *X*_*ij*_>*X*_*ji*_, viz. *i* imports more from *j* than what *i* exports to *j*.

Taking *S*_*i*0_ to the LHS of (5), rearranging and dividing through by *S*_*i*0_, we get the classic dynamical ‘epidemic’ equation (see, [58]) for tracking depletion or failure rates for a network system:

$$\mu_{it+1}=(1-\rho_{i})\mu_{it}+\sum_{j}\frac{{(X}_{ij}-X_{ji}{)}^{+}}{S_{i0}}\mu_{jt}.$$ (6)

In matrix notation,

$$U_{t+1}={\left[Diag(1-\rho_{i})+\theta \right]}^{t}U_{1}=Q^{t}U_{1}≅g\lambda_{max}^{t}(Q)v^{\#}.$$ (7)

Here, ***U***_*t +1*_ is a *Nx1* vector of vulnerability rates for each country and ***U***_*1*_ is a non-negative initial vector with at least some positive elements. ***Diag***(1−*ρ*_*i*_) is a *NxN* identity matrix with (1−*ρ*_*i*_) along the diagonal, and ***θ*** is a *NxN* positive matrix with elements composed of $\frac{{(X}_{ij}-X_{ji}{)}^{+}}{S_{i0}}$. As discussed in [25], the solution of the dynamical Eq in (7) is entirely governed by the maximum eigenvalue, *λ*_*max*_>0, of the ***Q*** matrix and its near term t = 1 solution is given by the associated (right) dominant or principal eigenvector centrality, ***v***^#^ which uses 1-norm, viz. $v^{\#}implies\;\sum_{i=1}^{N}v_{i}^{\#}=1.$ Note, the 1-norm, ‖*v*‖_1_, is the sum of absolute values of vector ***v***. Further, *g* in (7) is a positive constant and see [25] for how it contributes to the speed with which countries can deplete their stockpiles. Also note, the *λ*_*max*_ is invariant to the norm being used and remains the same for a matrix and its transpose. In contrast, eigenvectors ***v*** associated with *λ*_*max*_ are unique only up to a scalar. But when they are normalized as $v^{\#}=\frac{v}{{\left\|v\right\|}_{1}}$, we obtain *v*^*#*^ which is unique and whose elements add up to 1.

The significance of this framework is that when countries follow similar policies, when faced with conditions that deplete stockpiles, *S*_*i*0_, how extant trade networks will propagate these shocks can be assessed as an *ex ante* probability of shortfalls of net imports that depend on the first order and higher order interconnections between net importers and their trading partners. Strategies that relate to decreasing exports, *X*_*ji*_ from *i* to *j*, especially by those that remain dependent on imports from same net exporters, may not succeed in reducing their vulnerability by these activities due to the interconnections in the trade network to other countries that may do the same. Only on using the power iteration algorithm on matrix ***Q*** in (7) can the status of how countries fare in their self-sufficiency strategies be determined incorporating both the impact of their direct and indirect trading partners. Based on Perron-Frobenius Theorem, as ***Q*** is a real non-negative matrix, convergence is guaranteed to a real positive number for the maximum eigen-value, *λ*_*max*_(***Q***), and to the associated (right) principal eigenvector denoted by ***v***^#^ with non-negative eigenvector centrality values, *v*_*i*_^*#*^, for each node. Thus, the vector of vulnerability rates in (7) converges to the fixed point yielding the principal eigenvector (see, [25], Eqs (12)–(15))

$$v^{\#}=\frac{1}{\lambda_{max}}Q\;v^{\#}$$ (8)

$$where\;\lambda_{max}={\left\|Q\;v^{\#}\right\|}_{1}=\sum_{i=1}^{N}(\sum_{j\neq i}\frac{{(X}_{ij}-X_{ji}{)}^{+}}{S_{i0}}v_{j}^{\#}+(1-\rho_{i})v_{i}^{\#}).$$ (9)

Here, *λ*_*max*_ being evaluated as ‖***Q v***^#^‖_1_ in (9) as first shown in [25], with

$0{\leq v}_{i}^{\#}<1,\;\sum_{i=1}^{N}v_{i}^{\#}=1,$ these can be viewed as the probability of depletion of stockpiles and hence $v_{j}^{\#}$ in the first term in (9) adversely affects country *i’s* expected net imports from *j*. Thus, the first term in (9), which is ‖***Qv***^#^‖_1_ and denoted as *λ*_*max*_(***Q***), is the probability weighted average or expected shortfall of net imports of each country, $\sum_{i\neq j}^{N}{(X}_{ij}-X_{ji}{)}^{+}\;v_{j}^{\#},$ given as a percentage of its buffer, *S*_*i*0_, and aggregated for the system as a whole. The second term in (9) gives the probability weighted average of (1−*ρ*_*i*_) based on *i’s* own probability for depletion of stockpiles.

In other words, the vulnerability centrality for each country in (8) is related not just to the extent of its exposure to other countries for imports via the matrix ***Q***, but also to the vulnerability centrality of its trading partners. Further, the time invariant or near time solution (t = 1) in (7) (see, [58]) yields the eigenvalue equation for what will be termed the systemic vulnerability rates in (10) which is the product of *λ*_*max*_ which is common to all countries and the norm-1 eigenvector vulnerability centrality for each country $v_{i}^{\#}$:

$$U^{*}=\lambda_{max}\;v^{\#}=Q\;v^{\#}.$$ (10)

Where in (6), the thresholds *ρ*_*i*_ *= 0*, viz. there is no policy for the replenishment of stockpiles, *λ*_*max*_ (*Q*) = *λ*_*max*_ (***θ***)+1. This implies that if the stockpiles are not replenished at all viz. *ρ*_*i*_ = *0* for all *i* countries in (6), in the absence of other interventions, the system can be expected to suffer further shortfalls of net imports at the rate of *λ*_*max*_(***θ***) at each time step going forward (see, [25]). Indeed, with a policy of replenishment of stockpiles at the same rate of *ρ*>*0* for all countries in (6) implies that *λ*_*max*_ (***θ***)≤*ρ* is required to mitigate over time the expected shortfalls in a country’s net imports from trading partners in a similar predicament. What this means is that coordinated mitigation strategies based on the knowledge of *λ*_*max*_(*θ*) for the system as a whole, which [25] compared to the epidemic R number, is needed to combat the consequences of high import dependence for critical medical products. In contrast, uncoordinated export reduction and other ‘beggar my neighbour’ strategies can backfire. Note non-zero vulnerability centrality index for countries based on the right eigenvector centrality of the ***θ*** matrix follows for those countries which are *both* net importers from some countries while being net exporters to at least one of the remaining other countries. The model in (7), in the first instance gives vital topological information from the bilateral global trade network in terms of *λ*_*max*_ (***θ***) > 0 of how much the system as a whole can be expected to suffer in import shortfalls in the absence of any coordinated interventions.

This framework from [25] adds to the Google PageRank algorithm by [27,28]. [27] showed that PageRank corresponds to the principal eigenvector of the normalized link matrix of the Web. However, note the Google page rank bilateral matrix is positive stochastic in which every entry is positive, and each row adds up to 1. Hence, in the Google page rank model, the maximum eigenvalue, *λ*_*max*_, is 1. This is not the case in economic cascade models for global trade networks. Thus, the eigen-pair method also incorporates information from *λ*_*max*_ in addition to the eigenvector centralities. The product of *λ*_*max*_ (***θ***) and $v_{i}^{\#}$ when multiplied by the total buffers or stockpiles for each country for the medical product, *S*_*i*0_, can give the $-value of each country’s expected shortfalls, $\sum_{i\neq j}^{N}{(X}_{ij}-X_{ji}{)}^{+}\;v_{j}^{\#},$ from net imports brought about by the vulnerability centrality measures, $v_{j}^{\#}$, for the *j* countries vis-à-vis which *i* is a net importer. [25,26,42,59] use simulation exercises to show how different size buffers have to be built up based on the vulnerability centrality measures to reduce the systemic *λ*_*max*_ and also on the implications of different threshold values, *ρ*, for replenishing stockpiles when adopted by countries.

Due to a paucity of data on domestic production of the medical products for countries and also on their policies for stockpiling these products, we use the intra-regional trade data summed over the countries based on the regions they belong to as the regional buffer (viz. the term in the denominator of the bilateral net import variables in Eq (6)) to proxy the strategy of regional self-sufficiency. Thus, noting the regional imports equal and regional exports, we construct the ***θ***^*R*^ matrix (given in **S1 Appendix Section B** equation B.3) and directly apply the eigenvalue equation for the eigenvector centrality vulnerability indexes based on the assumption of regional self-sufficiency. This leads to the following regional self-sufficiency variant of Eq (10), which will be used to evaluate the extent to which a EU27 countries suffer an expected shortfall in net imports from all bilateral net exporter countries *j*, that belong to both intra and extra EU-27:

$$\lambda_{max}(\theta^{R})v^{EU\#}=\frac{1}{{RE}^{EU27}}\sum_{i=1}^{EU27}(\sum_{i\neq j}^{N}{(X}_{ij}-X_{ji}{)}^{+}\;v_{j}^{\#},$$ (11)

Here, the EU27 wide eigenvector centrality vulnerability index $v^{EU\#}=\sum_{i=1}^{EU27}v_{i}^{\#}$, and all EU27 countries have a common denominator equal to the EU27 intra-regional export/imports denoted by *RE*^*EU*27^ in the ***θ***^*R*^ matrix. This yields the $ value of the expected shortfall of net imports that EU27 can experience in relation to the assumed proxy for regional self-sufficiency from topology of the bilateral trade flows globally for each of the medical products. For this the product of the *λ*_*max*_ (***θ***^*R*^) ***v***^*EU*#^ in Eq (11) is multiplied by the $-value of EU27 intra-regional export/imports denoted by *RE*^*EU*27^:

$$\lambda_{max}(\theta^{R})\;v^{EU\#}{RE}^{EU27}\equiv \$Expected\;losses\;in\;net\;EU\;imports\;for\;a\;medical\;product.$$ (12)

## 5. Empirical network analyses for global and Intra-EU trade networks for critical medical products

### 5.1 Network statistics for Intra-EU trade network (2019, 2020, 2021)

First, we summarize some topological network statistics described above for global trade network and intra-EU trade networks. These network measurements are presented in the **Table 7**.

**Table 7:** Topological network statistics for Intra-EU and global gross trade export/import networks (Pre (2019), pandemic onset (2020) and adaptation year (2021)).

Descriptive statistics		Nodes	Edges	Clustering Coefficient	Assortativity Coefficient (Export-based)	Assortativity Coefficient (Import-based)	Reciprocity	Density
Intra-EU trade	Face Mask 2019	27	617	0.977	-0.065	-0.080	0.901	0.879
	Face Mask 2020	27	648	0.985	-0.056	-0.058	0.923	0.923
	Face Mask 2021	27	628	0.987	-0.057	-0.062	0.898	0.895
	Ventilator 2019	27	392	0.915	-0.088	-0.122	0.658	0.558
	Ventilator 2020	27	237	0.894	-0.087	-0.146	0.321	0.338
	Ventilator 2021	27	373	0.875	-0.108	-0.139	0.654	0.531
	PPE 2019	27	283	0.767	-0.101	-0.107	0.629	0.405
	PPE 2020	27	281	0.768	-0.134	-0.138	0.633	0.400
	PPE 2021	27	280	0.809	-0.095	-0.089	0.607	0.399
	Sanitizer 2019	27	478	0.935	-0.093	-0.122	0.778	0.681
	Sanitizer 2020	27	588	0.975	-0.065	-0.077	0.878	0.838
	Sanitizer 2021	27	533	0.964	-0.080	-0.099	0.826	0.759
Global trade network	Face Mask 2019	208	6254	0.837	-0.037	-0.067	0.491	0.145
	Face Mask 2020	208	7353	0.862	-0.024	-0.065	0.521	0.171
	Face Mask 2021	209	6664	0.853	-0.029	-0.065	0.475	0.153
	Ventilator 2019	203	3221	0.675	-0.094	-0.084	0.343	0.079
	Ventilator 2020	203	2258	0.705	-0.082	-0.087	0.183	0.055
	Ventilator 2021	203	3554	0.652	-0.110	-0.089	0.350	0.087
	PPE 2019	180	934	0.477	-0.047	-0.007	0.353	0.029
	PPE 2020	185	1087	0.533	-0.032	-0.021	0.362	0.032
	PPE 2021	180	1041	0.516	-0.050	-0.042	0.330	0.032
	Sanitizer 2019	206	2994	0.722	-0.096	-0.092	0.305	0.071
	Sanitizer 2020	208	4031	0.787	-0.060	-0.045	0.380	0.094
	Sanitizer 2021	208	3621	0.735	-0.102	-0.086	0.348	0.084

Source: Authors’ calculation based on gross export or import matrix, as appropriate, for each product built with the data from UN Comtrade database. Note the maximum number of edges in the intra-EU network is 27 x 26 = 702; this number for the global networks ranged from 32,220 for PPE to 43,056 for face mask in, for instance in 2019.

When we compare edges from 2019 to 2020 for intra-EU trade network, an increase is observed in the number of edges for face mask and hand sanitizer implying that EU 27 countries diversified their intra-EU exports. In contrast, the number of edges seems almost static for PPE, while there is a severe decline in the number of edges for medical ventilators. This situation can also be observed in network density coefficient that represents connectivity for overall network. Accordingly, density coefficient for intra-EU trade network increases from 2019 to 2020 for face mask and sanitizer while it seems stable for PPE. However, a severe decline in this connectivity statistic is observed for ventilator from 0.56 in 2019 to 0.34 in 2020. When it comes to clustering, it is observed that there is an increase for face mask, PPE and sanitizer, meaning that the network become more transitive from 2019 to 2020 generally. That is, any two of country *i’s* neighbours *j* from which it is exporting to, also have such a relationship. However, clustering coefficient decreases for medical ventilator from 2019 to 2020, meaning that the network become less transitive in 2020 and neighbour countries in the intra-region trade of this product becomes less connected. Similarly, reciprocity indicator increases for face mask, PPE and sanitizer from 2019 to 2020, implying that for (*i*,*j*) pairs of countries, they both import and export each of these medical products while it decreases for medical ventilator. This indicates that EU27 countries can rely on one another to meet their needs for face mask, PPE and sanitizer. However, the same cannot be said for ventilator. For example, the export trade matrix shows that Germany exports ventilators to all other EU-26 countries in 2020, but it imports from only 12 EU countries. From 2020 to 2021, with the adaptation process, density and reciprocity values decrease somewhat for face mask, sanitizer and PPE while it increases for ventilator. It can be inferred that it takes much more time for regional supply chains to adapt to increases in domestic demand in all countries for high-tech products like ventilators with the result of more EU countries increasing production and stepping up exports. Finally, it is observed that assortativity coefficient for all products examined is negative in all years, meaning that core-periphery structure exists in all of these intra-region trade networks. In core-periphery structure, there exist highly connected core countries and also peripheral countries that are connected to the core. The periphery countries are not connected to one another. Hence, it can be thought as an indication of dominance of a few core countries in the network.

### 5.2 Network statistics for global trade network relating to EU 27 countries

Compared to the EU 27 regional networks for the 4 medical products, the global networks for them are sparse with the greatest network density for face mask not exceeding 17%. However, we see the same upward trend in 2019–2020 in the number of edges (the number of trade relations) for the global trade of the medical products in **Table 7** for all medical products other than for the ventilator which suffers a sharp decrease. We also observe a decline in reciprocity and density values of the ventilator trade networks from 2019 to 2020. These results confirm that countries could not rely on one another to meet their demand for ventilators in the first year of the pandemic. In the 2021 adaptation year of the pandemic, it is observed that these values for ventilators rise and get ahead of the values of 2020. These findings indicate that the global supply chain is not resilient in the face of an immediate surge of demand of such a complex medical product in an emergency, while it can adjust itself over time. Another inference from these findings is that the global supply chain is capable of adjusting itself to changing conditions for low-tech products much more than it can for a high tech one. Increasing values of edges, reciprocity and density are observed in the 2020 pandemic year for face mask, PPE and hand sanitizer, and these values decrease in the adaptation year as more self-sufficiency kicks in. Finally, it is seen that all networks display disassortative structure, meaning that core-periphery structure exists in all of these networks.

As a final topological property, we also examined the degree/strength distribution of these networks in order to see if diversification of imports is pursued as a trade strategy to mitigate risks from overspecialization and concentration of global production. The results are presented in **Table 8**.

**Table 8:** Degree distribution of export matrix analysis for intra-EU and global trade networks (for 2019, 2020 and 2021).

Measurements		Skewness	Kurtosis	Alpha, α	KS* statistics	p-value
Intra-EU trade	Face Mask 2019	3.094	13.168	1.812	0.153	0.897
	Face Mask 2020	3.358	15.094	2.896	0.105	0.999
	Face Mask 2021	2.958	12.149	1.875	0.129	0.954
	Ventilator 2019	3.050	11.967	2.205	0.169	0.988
	Ventilator 2020	3.050	10.662	1.242	0.179	0.762
	Ventilator 2021	2.941	10.936	1.319	0.128	0.866
	PPE 2019	1.953	6.390	1.255	0.164	0.542
	PPE 2020	1.681	5.008	1.609	0.205	0.597
	PPE 2021	3.088	13.151	2.754	0.137	0.999
	Sanitizer 2019	3.216	13.907	1.512	0.143	0.833
	Sanitizer 2020	3.078	13.044	1.664	0.120	0.936
	Sanitizer 2021	2.688	10.573	1.406	0.173	0.524
Global trade network	Face Mask 2019	12.922	178.114	1.552	0.154	0.228
	Face Mask 2020	14.234	204.392	1.970	0.053	0.999
	Face Mask 2021	13.743	194.938	1.977	0.084	0.996
	Ventilator 2019	6.096	43.770	1.382	0.135	0.494
	Ventilator 2020	5.864	39.342	1.351	0.168	0.509
	Ventilator 2021	5.138	29.839	1.241	0.140	0.109
	PPE 2019	5.955	42.052	1.543	0.135	0.753
	PPE 2020	6.092	48.282	3.201	0.110	0.999
	PPE 2021	5.081	30.129	1.348	0.095	0.693
	Sanitizer 2019	6.293	48.249	1.734	0.102	0.922
	Sanitizer 2020	11.087	140.773	1.739	0.095	0.845
	Sanitizer 2021	5.776	38.828	1.838	0.129	0.878

Source: Authors’ calculation. K-S statistic stands for Kolmogorov-Smirnov statistic, see Clauset et.al (2009).

Based on the p-value of KS statistic in **Table 8** that is greater than 0.05 for all networks, the null hypothesis referring to presence of power-law is accepted [41]. The power-law distribution of the global export matrix compared to that for intra-EU, for each of the medical products, indicates that the global network system is directed by fewer core nodes with high out degree (export) strength. Within this framework, examination of α is informative since it is also related to the number of super-nodes. A higher value of α leads to a lower probability of super-nodes in the network [54]. Accordingly, both for the intra EU trade network and for the global trade network, there is an increase of α for face mask, PPE and sanitizer from 2019 to 2020 while there is a decrease for ventilator from 2019 to 2020. Thus, an increasing value of α for face mask, PPE and hand sanitizer indicates diversification in this network of exporters diluting the impact of a few large exporters. The case of Chinese dominance in face masks exports in 2019, though somewhat mitigated in 2020 in terms of the power law exponent α, is captured by the very high kurtosis of over 200 observed in the degree distribution. The case of medical ventilator both at the global and intra EU level in 2020 show that the high tech components in its manufacture has resulted in countries specializing in its production and export rather than result in wide spread diversification observed in the other less sophisticated medical products. In 2021, intra EU does a better job at diversification of exports for ventilator and PPE than what is found in the global network.

## 6. Empirical results: Network vulnerability centrality and regional self-sufficiency index

This section gives results for the Markose-Giansante eigen-pair method developed in **Section 4.2** for the global trade cascade model for the 4 medical products from the perspective of evaluating EU-27 regional self-sufficiency. For this EU27 centric model, we include only the EU27 countries and also top 5 extra-EU net exporter countries to EU27’s imports for the 4 medical products. Depending on the medical product, these include a total of 44 countries with China, Vietnam, Thailand, Singapore, Hong Kong, Malaysia constituting Asian region; Tunisia, Morocco, Turkey, representing Africa; US, UK, Australia, Norway, New Zealand and Switzerland, categorized as non-EU Western countries (see, **S1** Appendix Section **A**). To compute the EU27 expected net import shortfalls in each of the years for this trade network system, we first report the *λ*_*max*_ (***θ***^*R*^) for the matrix ***θ***^*R*^ which represents the so-called R number [25] for the 44 country trade network that gives the total expected shortfall in net imports for the countries as a percentage of the respective intra-regional imports of the countries. As explained in Eq (11), this is multiplied with the EU27 vulnerability centrality measure to determine the EU27 share of *λ*_*max*_ (***θ***^*R*^). This distinction between system wide *λ*_*max*_ (***θ***^*R*^) and intra-regional or country level eigenvector vulnerability centrality is useful to identify the source of expected shortfalls in net imports.

The EU27 vulnerability centrality index in **Table 9B** for face masks in 2019 is at 91% of the system wide ***λ***_***max***_ (***θ***^***R***^) of only 18%, is a case that illustrates this point. The EU27 import vulnerability centrality index is the highest for face masks of all medical products with the massive EU % share of imports from China alone increasing from 25% to 70% in 2021 (see, **Table 3**). This vulnerability centrality is reduced to 80% by 2021 as EU27 substitutes extra EU imports with intra EU % share of total EU imports and this increases from 20.1% to 46% (see, **Table 5**). Likewise, EU27 vulnerability centrality index in **Table 9B** from import reliance on extra EU countries is evidenced for PPE in all 3 years, spiking in 2020 pandemic year at 82%. However, as the system wide global expected shortages as indicated in *λ*_*max*_ (*θ*^*R*^) for face masks and PPE in **Table 9A** are relatively low, especially in 2020 when there was a massive increase in global exports, respectively, from China and Thailand, the vulnerability shares for EU27 given as the product of *λ*_*max*_ (*θ*^*R*^) and *v*^*EU*#^ (see, square brackets in **Table 9B**) are substantially low and in line with that for hand sanitizer for which EU27 has very high inherent self-sufficiency with low eigenvector vulnerability centrality. Ventilator is one where both systemwide *λ*_*max*_ (*θ*^*R*^) and the inherent vulnerability of EU27 are high in 2019 and 2020. In 2021 both are mitigated. Finally, as shown in Eq (12), the $-value of EU27 expected import shortfalls (see **Table 9D**) is obtained in terms of the common measure for regional buffers which is proxied by the EU27 intra-regional exports/imports given in **Table 9C**. In keeping with the upward direction in 2021 for face mask and PPE in both global *λ*_*max*_ (*θ*^*R*^) and the EU27 share of expected shortfall of net imports, *λ*_*max*_(*θ*^*R*^)*v^*EU*#^, only for these products does the dollar value of the latter go up, respectively to about $606 mn and $7.95 mn compared to the values in 2019 and 2020.

**Table 9:** Systemic expected shortfalls in net imports of each of the 4 medical products for the 44 country EU-centric global network.

**A. Maximum eigenvalues: *λ***_***max***_ **(*θ***^***R***^**) in Eq (9) for the *θ***^***R***^ **global trade network matrix described in SI Appendix Section B equation (B.3)**														
			Face mask			Ventilator		PPE			Hand sanitizer			
2019			0.19			0.34		0.23			0.44			
2020			0.06			0.37		0.19			0.15			
2021			0.22			0.26		0.27			0.11			
**B. $ EU27 Norm-1 Vulnerability Centrality Index *v*^*EU*#^ Term in square brackets [*λ***_***max***_ **(*θ***^***R***^**)*v***^***EU*#**^**] EU Share of Global expected Shortfalls (see Eq (11))**														
	Face mask				Ventilator				PPE			Hand sanitizer		
2019	0.91 [0.17]				0.60 [0.20]				0.65 [0.15]			0.33 [0.14]		
2020	0.80 [0.05]				0.58 [0.22]				0.82 [0.15]			0.62 [0.09]		
2021	0.78 [0.17]				0.61 [0.16]				0.66 [0.18]			0.18 [0.02]		
**C. $ millions Intra- Regional export/import of EU27 Proxy for Regional Self-Sufficiency (RE** ^EU27^ **)**														
	Facemask						Ventilator			PPE				Hand sanitizer
2019	1,895						1,120			35.32				726
2020	4,945						1,196			50.28				1,457
2021	3,560						1,453			43.75				1,061
**D. $ millions EU27 Expected Shortfall in Imports (*λ*_*max*_ (*θ*^*R*^)*v*^*EU*#^ REEU27) See Eq (12)**														
		Face mask		Ventilator				PPE					Hand sanitizer	
2019		326.93		232.05				5.31					101.64	
2020		238.15		257.10				7.70					131.13	
2021		606.55		226.80				7.95					21.22	

**Fig 1:**
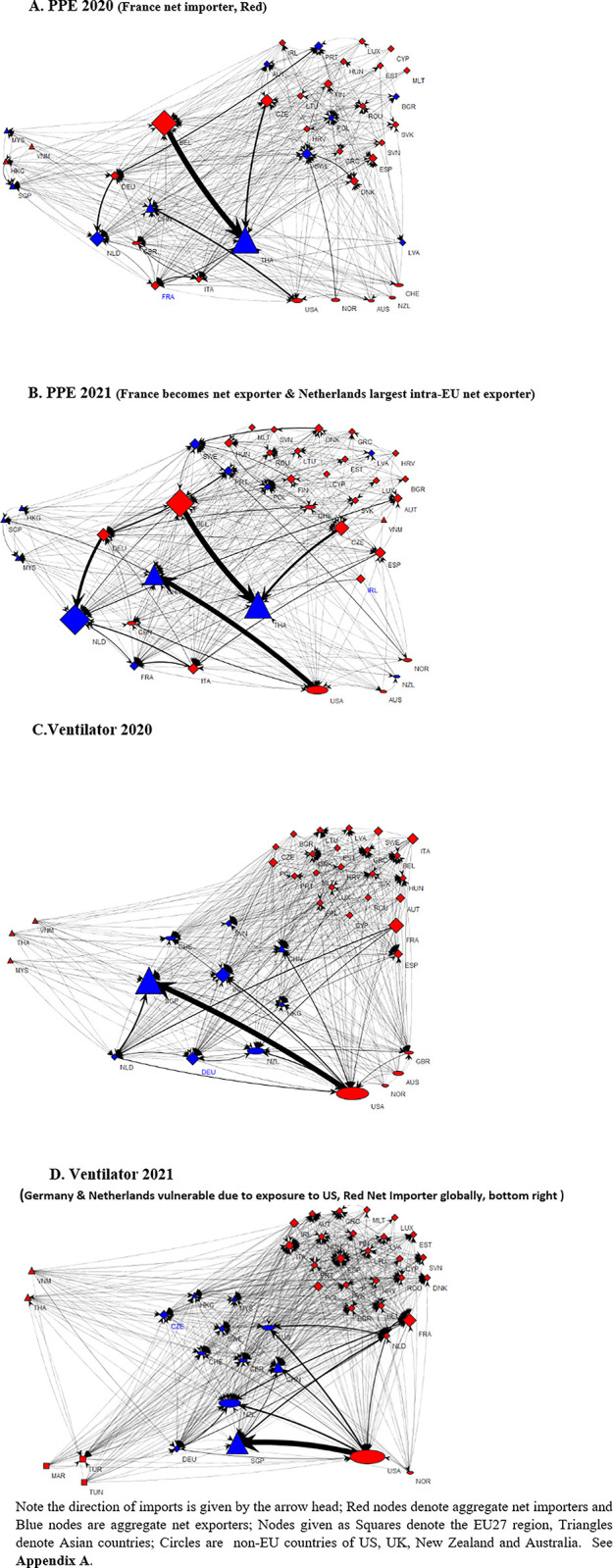
Selected global trade networks for PPE and ventilator in 2020 and 2021.

We now turn to the vulnerability centrality measures based on Eq (8) for each of the top 5 EU27 countries and the top 5 extra-EU countries, which are given in **Table 10. Table 10** gives a summary of which countries succeeded in mitigating their import vulnerability by 2021 following data in **Table 5** that gives evidence for large shifts away from extra EU imports to intra EU self-sufficiency. These analyses are accompanied by the network graphs in **Fig** 1, which illustrate the point that vulnerability centrality of a country depends on their bilateral net importer status with other highly vulnerable net importers. In **Table 10**, the rank order from highest to lowest is given in terms of the 2020 centrality measures and in each year the darkest shade of the heat map identifies countries with the highest eigenvector vulnerability centrality. Thus, for face mask, while Spain started off as being the most vulnerable EU country in pre-pandemic 2019, France takes over in first place for 2020 and 2021. **Table 10** shows that France was worse off in 2021 than in 2020. In contrast, other EU countries with a small exception of Italy reduce their vulnerability by 2021. In extra-EU countries, countries like Great Britain, Switzerland and US do not succeed in reducing their vulnerability to global trade networks in face masks by 2021.

**Table 10:** Vulnerability centrality index based on norm-1 right eigenvector centrality of Top 5 EU27 and top 5 non-EU countries.

Vulnerability Centrality index for top 5 EU countries							
Face mask				Ventilator			
Countries	2019	2020	2021	Countries	2019	2020	2021
FRA	0.2982	0.2871	0.2886	NLD	0.0981	0.1564	0.1334
ESP	0.4619	0.1997	0.1423	FRA	0.0759	0.1227	0.1089
BEL	0.0531	0.1013	0.0366	DEU	0.0863	0.0702	0.0885
IRL	0.0122	0.0948	0.0643	ITA	0.0305	0.0596	0.0341
ITA	0.0263	0.0260	0.0586	ESP	0.0318	0.0372	0.0489
PPE				Hand sanitizer			
Countries	2019	2020	2021		2019	2020	2021
FRA	0.1323	0.3871	0.0694	IRL	0.0304	0.1607	0.0020
ITA	0.0852	0.1277	0.0305	BEL	0.1495	0.1313	0.0991
IRL	0.0017	0.0874	0.2763	NLD	0.0606	0.0870	0.0183
ESP	0.0642	0.0771	0.0191	ESP	0.0181	0.0354	0.0023
BEL	0.1774	0.0276	0.1207	FRA	0.0067	0.0301	0.0072
Vulnerability index for top 5 extra-EU countries							
Face mask				Ventilator			
Countries	2019	2020	2021	Countries	2019	2020	2021
AUS	0.0311	0.1174	0.0424	USA	0.1889	0.1844	0.1738
USA	0.0169	0.0292	0.0272	GBR	0.0744	0.0917	0.0022
CHE	0.0139	0.0234	0.0284	AUS	0.0023	0.0578	0.0113
NOR	0.0026	0.0122	0.0069	THA	0.0204	0.0202	0.0385
GBR	0.0045	0.0084	0.0648	CHE	0.0104	0.0135	0.0179
PPE				Hand sanitizer			
Countries	2019	2020	2021	Countries	2019	2020	2021
GBR	0.1776	0.0927	0.2057	AUS	0.1395	0.0921	0.1437
CHE	0.0192	0.0386	0.0086	NZL	0.0290	0.0665	0.0652
USA	0.0542	0.0212	0.0718	USA	0.1556	0.0513	0.0816
NOR	0.0044	0.0111	0.0014	THA	0.0363	0.0342	0.0956
HKG	0.0229	0.0063	0.0002	VNM	0.0444	0.0265	0.1558

For PPE, from **Table 10**, France again stands out as the most vulnerable in 2020 in EU27 as net importer (bottom left in **Fig 1A**) from net importers (red nodes in **Fig 1A**) Belgium, Germany, Great Britain, USA and Italy. Though Belgium has an outsized import dependence on Thailand (largest blue triangle in **Fig 1A**), what is interesting is that as Thailand is a net exporter and has low to zero import vulnerability, it does not contribute to Belgium’s vulnerability centrality. In contrast, Great Britain dominates in terms of vulnerability centrality for PPE among the non-EU countries and France is a net importer from Great Britain. **Table 10** and **Fig 1B** for 2021 show that France reduces its vulnerability in PPE by switching to being a net exporter in aggregate (France node becomes blue) and Ireland becomes most vulnerable, again because of import dependence on Great Britain. For PPE, Netherlands becomes the biggest net exporter hub servicing intra-EU countries like Italy, France, Germany, Ireland and Belgium. However, as noted from **Table 9B**, EU27 as whole does not reduce its vulnerability index for PPE in 2021 given by the figures in square brackets. This is also the case of face masks.

**Table 11** gives the $ value of the vulnerability experienced by the top 5 EU27 countries for PPE in terms of expected shortfalls due to their counterparties suffering similar shortfalls and hence unable to fulfil their exports. Thus in 2020 for PPE, this is estimated to be $3.62 mn for France, which is reduced to $0.82mn in 2021 while for that year Ireland faces expected shortfalls in net imports as high as $3.28 mn and Belgium of $1.44 mn. Both these countries had expected net import shortfalls of under a million in 2020 as shown in **Table 11**.

**Table 11:** Top 5 countries: Expected shortfalls in net imports ($ millions) for PPE and ventilators, 2020 and 2021 using Eq (11); [*vi#*] square brackets give the norm-1 eigenvector centrality for country *i*.

Top 5 vulnerable countries	PPE 2020 ***λ***_***max***_ **(*θ***^***R***^**) =** 0.19	Top 5 vulnerable countries	PPE 2021 ***λ***_***max***_ **(*θ***^***R***^**) =** 0.27
France	$3.62 mn	**Ireland**	**$3.29 mn**
	[0.39]		**[0.276]**
Italy	$1.19 mn	**Belgium**	**$1.44 mn**
	[0.13]		**[0.12]**
Ireland	$0.817	France	$0.83 mn
	[0.09]		[0.07]
Spain	$0.721	Czechia	$0.58 mn
	[0.08]		[0.048]
Belgium	$0.258	Germany	$0. 37 mn
	[0.028]		[0.031]
Top 5 vulnerable countries	Ventilator 2020 ***λ***_***max***_ **(*θ***^***R***^**) =** 0.37	Top 5 vulnerable countries	Ventilator 2021 ***λ***_***max***_ **(*θ***^***R***^**) =** 0.26
Netherlands	$68.93 mn	Netherlands	$49.63mn
	[0.16]		[0.13]
France	$54.08 mn	France	$40.51mn
	[0.12]		[0.11]
Germany	$30.93 mn	**Germany**	**$32,92 mn**
	[0.07]		[0.09]
Italy	$26.27mn	**Spain**	**$18.19 mn**
	[0.06]		**[0.05]**
Spain	$16.38 mn	Romania	$13.65 mn
	[0.04]		[0.05]

In the case of ventilators, **Table 10** shows that the vulnerability of the leading EU producers, viz. Netherlands and Germany, increases and remain heightened in years 2020 and 2021. Intra-EU, France ranks 2^nd^ in terms of vulnerability after Netherlands while extra-EU, the USA is most vulnerable globally throughout the pre-pandemic and pandemic years for ventilators. The vulnerability of Netherlands and Germany, despite being aggregate net exporters in 2020, can be explained from **Fig 1B**. They import from Singapore, US and from one another with US being the highest ranked vulnerability central non-EU country. From **Table 10**, the latter is due not so much because of the large import dependence of US on Singapore, but due to imports from UK, Australia and Switzerland which are top 5 non-EU in terms of vulnerability centrality. **Fig 1C** shows how in 2021, Netherlands becomes an aggregate net importer (node colour is red) and continues being most vulnerable intra-EU country while UK reduces its vulnerability by becoming an aggregate net exporter in 2021 (node turns blue). **Table 11** shows that the $-value of expected shortfalls for the top vulnerability central EU country, the Netherlands, for ventilator are quite substantial in the order of $68.93 mn in 2019 and $49.63mn in 2020. For the top 5 EU27 countries this expected shortfall falls substantially except for Germany and Spain where it goes up in 2021 by $2mn compared to 2020.

For hand sanitizers, **Table 10** shows that among the EU27, Ireland, Belgium and Netherlands show the greatest vulnerability while globally Australia tops the list. In 2021, with the inherent self-sufficiency of EU27 for this product, with Germany and Spain stepping up and becoming large net exporters serving the intra-EU27, the 2020 vulnerability of Ireland, Belgium and Netherlands is almost fully mitigated.

As discussed, such a handle on potential systemic shortfalls given by the eigen-pair, *λ*_*max*_ (*θ*^*R*^) and the eigenvector centrality ***v***^***#***^, from the topology of the trade network in terms of the ratio of bilateral net imports and intra-regional imports to proxy regional self-sufficiency, is useful in crisis management. The product of *λ*_*max*_ (*θ*^*R*^) and the eigenvector vulnerability centralities for each country, is a good tool to target *ex ante* country level expected import shortfalls in terms of regional self-sufficiency. However, given the domestic shortfalls experienced within countries, the intra-regional export data used to proxy for domestic self-sufficiency is not suitable and data on domestic production and stockpiles for critical medical products should be collected and made publicly available.

## 7. Conclusion and future work

The pandemic has led to concerns about Global Supply Chains (GSCs) regarding their resilience against shocks since they failed to meet the sudden surge in demand for vital medical goods and raw materials required to produce these goods. Overspecialisation, geographical concentration of production and single sourcing were key characteristics of GSCs, shaped by low-cost considerations that favoured certain narrow objectives of influential economic actors [12]. Topologically this implies power law distributions in the global trade networks found in the pre pandemic era as indicated in **Section 5 Table 8**. Clearly, resilience and the efficacy of the cross-border trade network system during emergencies were not major considerations in the globalization process of production networks. **Section 5 Table 8** shows how with the onset of the pandemic, with the exception of ventilator, in the global trade networks for the other 3 medical products there is an increase of the power law distribution parameter α from 2019 to 2021. This result indicates that the number of super-nodes in the net import trade network of these products decreased during the pandemic as specialization by countries fell and countries diversify their sources for imports while they also diversify their domestic capabilities.

In the face of these drawbacks of extant global trade networks with the onset of Covid-19, the resilience of healthcare systems has become the focus of policy and the area of many academic studies that we have reviewed. The critical nature of the medical products in the fight against the Covid-19 pandemic motivated policy initiatives for reshoring the globally distributed supply chains for the EU region. However, it is clear that there is as yet no modelling framework in the literature to quantitively assess the effectiveness of policy implementations concerning onshoring and regionalisation of globally distributed production networks and also of restricting exports in the pandemic period.

The network eigen-pair methodology in **Section 4** based on [25,42], gives wide scope for quantitative assessment of vulnerabilities from import dependency and policy drives to increase self-sufficiency by increasing stockpiles of critical medical products and diverting exports for domestic consumption. The latter is baked into the model where vulnerability of a country is driven by exposure in terms of net imports from bilateral trade with net exporters. The efficacy of export restriction and increased buffer strategies must be assessed within fixed point solutions for interconnected systems like trade networks involving maximum eigenvalue and principal eigenvector centrality measures that are based on systemic characteristics. Thus, in addition to Google PageRank style vulnerability centralities for individual countries arising from their import dependence for critical medical supplies, the maximum eigenvalue (λ_max_) in Eq (9) can give estimates for expected shortfalls from net imports as % of regional or country buffers for the trade network as a whole. This corresponds to the so-called R-number for systemic risk of epidemics [25].

**Table 9** shows how the overall massive expansion of global exports and intra-EU regional imports helped reduce the system wide *λ*_*max*_ (*θ*^*R*^), at the onset of Covid-19 in 2020 for face masks, PPE and hand sanitizer. Adapting production facilities for low-tech products seems to be easy, however it is more challenging for the more complex and high-tech product as in medical ventilators. Thus, the case of ventilators with the increased *λ*_*max*_ (*θ*^*R*^) in 2020, in **Table 9A**, reflects the problems of increasing both intra-EU and global production and trade in it in the short run. Interestingly, in the adaptation year of 2021, ventilator and hand sanitizers show a downward trajectory for *λ*_*max*_ (*θ*^*R*^) and also in lower dollar values in expected EU27 regional shortfalls (**Table 9D**). However, the extra EU dependency for face masks and raw materials for PPE for the EU27 countries combined with self-sufficiency strategies contribute to an increase in *λ*_*max*_ (*θ*^*R*^) and also greater EU29 vulnerability (**Table 9B**) and also higher expected shortfall $ values for these two medical products (**Table 9D**).

Finally, results in **Table 10** using the eigenvector vulnerability centrality measure for individual countries show which EU-27 and extra EU countries took the brunt of expected net import shortfalls in 2019, 2020 and 2021 for each of the 4 medical products. **Table 10** and **Fig 1** highlight the fixed point result here for import vulnerability centrality in the case of Germany and Netherlands, for example, for ventilators. This persists in 2021 despite these countries being the major intra-EU27 net exporters and this is because of their import dependence on extra-EU countries like US and UK, which have high vulnerability centrality.

In [26,59] extensive analyses have been done on improving the resilience of network systems prone to failure propagation using the eigen-pair method. The important connection between the so called *λ*_*max*_ for the specially constructed trade network matrix and the thresholds at which stockpiles or buffers of critical products are targeted not to go below, needs further analysis. Simulation and agent-based modelling for interconnected systems (see [60]) and better data regarding domestic production and stockpiles of critical medical products in the different countries are needed to bring this research agenda to full fruition.

## Supporting information

S1 Appendix Regional classification for 44 countries that include top 5 non-EU 27 exporters to EU-27 countries. (DOCX)

## Abbreviations

AI: Artificial Intelligence
FEMA: Federal Emergency Management Agency
GSC: Global Supply Chain
KS: Kolmogorov-Smirnov
PPE: Personal Protective Equipment
UN: United Nations
WTO: World Trade Organization
