# Supplementary material for: EU27 regional trade networks for medical products in fight against Covid-19 pandemic: Quantifying vulnerability and self sufficiency in critical inputs
Source: PLoS One. 2024 Feb 23;19(2):e0297748. doi: 10.1371/journal.pone.0297748 (PMC10889623; doi:10.1371/journal.pone.0297748)
Supplement: S1 Appendix — (DOCX) [file pone.0297748.s001.docx]

**SI Appendix A Table. Regional classification for 44 countries that include top 5 non-EU 27 exporters to EU-27 countries**

| EU27 countries | Austria, Belgium, Bulgaria, Cyprus, Czechia, Germany, Denmark, Spain, Estonia, Finland, France, Greece, Croatia, Hungary, Ireland, Italy, Lithuania, Luxembourg, Latvia, Malta, Netherlands, Poland, Portugal, Romania, Slovakia, Slovenia, Sweden |
| --- | --- |
| Non-EU Western countries | Australia, Switzerland, United Kingdom, Norway, New Zealand, USA |
| Asian countries | China, Hong Kong, Malaysia, Singapore, Thailand, Vietnam |
| Other/African Countries | Morocco, Tunisia, Turkey |

**S2 Appendix B: Regional Self-Sufficiency Net Import Matrix**

Starting with the gross import matrix X in , which is a $N\times N$ directed and weighted network with elements $X_{ij}$ denotes the import of country i from country j.

Then, we obtained netted import matrix (M) by applying $X-X'$:
$M=\left[ \right]$ (B.1)

By taking only positive net import values (the value is zero if $(X_{ij}-X_{ji})<0$), we obtained $M^{+}$ netted import matrix which is skew symmetric and contains only non-negative elements , viz. if *m^+^_ij_ >0 then m^+^_ji_ =0 .*

$$M^{+}=\left[ \right] (B.2)$$

Then, we divided each positive net import values of country *i* from *country j in* $M^{+}$ matrix by intra- regional export/import (*RE_i_* ) values that country *i*  belongs to. This yields $\Theta$*^R^* matrix:

$\theta^{R}=\left[ \right]$ (B.3)

Thus, note for example that all EU-27 countries, which occupy 27 rows of the 44 country $\Theta$ matrix have the same intra-regional exports , *RE_i_* , equal to the intra-EU 27 exports/imports, in the denominator of the $\Theta$*^R^* matrix in (B.3). This yields the results in equations (11) and (12).
